# Lysine demethylase KDM2A inhibits TET2 to promote DNA methylation and silencing of tumor suppressor genes in breast cancer

**DOI:** 10.1038/oncsis.2017.71

**Published:** 2017-08-07

**Authors:** J-Y Chen, C-W Luo, Y-S Lai, C-C Wu, W-C Hung

**Affiliations:** 1 National Institute of Cancer Research, National Health Research Institutes, Tainan, Taiwan; 2 Department of Pathology, Kaohsiung Medical University Hospital, Kaohsiung Medical University, Kaohsiung, Taiwan; 3 Cancer Center, Kaohsiung Medical University Hospital, Kaohsiung, Taiwan; 4 Graduate Institute of Medicine, College of Medicine, Kaohsiung Medical University, Kaohsiung, Taiwan

## Abstract

The coupling between DNA methylation and histone modification contributes to aberrant expression of oncogenes or tumor suppressor genes that leads to tumor development. Our previous study demonstrated that lysine demethylase 2A (KDM2A) functions as an oncogene in breast cancer by promoting cancer stemness and angiogenesis via activation of the Notch signaling. Here, we demonstrate that knockdown of KDM2A significantly increases the 5′-hydroxymethylcytosine (5′-hmc) level in genomic DNA and expression of tet-eleven translocation 2 (TET2) in various breast cancer cell lines. Conversely, ectopic expression of KDM2A inhibits TET2 expression in KDM2A-depleted cells suggesting TET2 is a transcriptional repression target of KDM2A. Our results show that KDM2A interacts with RelA to co-occupy at the TET2 gene promoter to repress transcription and depletion of RelA or KDM2A restores TET2 expression. Upregulation of TET2 in the KDM2A-depleted cells induces the re-activation of two TET downstream tumor suppressor genes, epithelial cell adhesion molecule (EpCAM) and E-cadherin, and inhibits migration and invasion. On the contrary, knockdown of TET2 in these cells decreases EpCAM and E-cadherin and increases cell invasiveness. More importantly, TET2 expression is negatively associated KDM2A in triple-negative breast tumor tissues, and its expression predicts a better survival. Taken together, we demonstrate for the first time that TET2 is a direct repression target of KDM2A and reveal a novel mechanism by which KDM2A promotes DNA methylation and breast cancer progression via the inhibition of a DNA demethylase.

## Introduction

DNA methylation and histone modifications are two major epigenetic regulatory processes that control gene expression, genomic stability, imprinting and chromosome structure.^1, 2, 3^ DNA methylation, the addition of the methyl group to the cytosine of the CpG dinucleotides, is mainly catalyzed by three DNA methyltransferases (DNMTs) including DNMT1, DNMT3A and DNMT3B, and is strongly associated with gene repression. DNA methylation has been considered to be an extremely stable epigenetic marker until the identification of the tet-eleven translocation (TET) gene family.^4, 5^ This family contains three members including TET1, TET2 and TET3, and the encoded proteins are Fe^2+^- and α-ketoglutarate-dependent dioxygenases which can hydrolyze 5′-methylcytosine (5’-mc) to 5’-hydroxymethylcytosine (5′-hmc) and finally erase the methyl group from the CpG dinucleotides. Therefore, the TET enzymes function as DNA demethylases which antagonize DNMT-mediated DNA methylation and gene repression. Compared to DNA methylation, histone modifications are complex and the modifications like methylation, phosphorylation, ubiquitination, sumoylation and so on, are catalyzed by many enzymes that add or remove the functional groups on specific residues of the histone proteins dynamically to generate the so called ‘histone code’.^6, 7^

The crosstalk between DNA and histone methylation in the regulation of gene transcription was firstly suggested by the observation that DNA methylation is frequently co-existed with the methylated lysine 9 of histone H3 (H3K9), a repression histone marker.^8, 9^ This hypothesis was further supported by a study showing that the mouse embryonic stem cells lack Suppressor Of Variegation 3-9 Homolog 1 (Suv39H1) and Suv39H2, the histone methyltransferases responsible for the tri-methylation of H3K9, exhibited a significant reduction of DNA methylation.^10^ How the methylation of DNA and histone is co-regulated is an important issue in gene regulation. Currently, three mechanisms have been proposed for the co-regulation. Firstly, histone methyltransferases may directly interact with DNMTs to form a functional complex and work together to coordinate DNA and histone methylation simultaneously. For example, two H3K9 methyltransferases G9a and GLP which catalyze the mono- and di-methylation of H3K9 have been shown to interact with DNMT3A and 3B to enhance *de novo* DNA methylation.^11^ Secondly, histone demethylases can also bind with DNMTs to affect epigenetic modification. Brenner *et al.* showed that the increased binding of KDM1A to DNMT1 during the S-phase of cell cycle may play a role in the control of DNA replication.^12^ Third, histone demethylases may modulate the enzymatic activity or protein stability of DNMTs to alter DNA methylation.^13^

Lysine demethylase 2A (KDM2A) was firstly identified as a novel Jumonji-C (JMJC) domain-containing proteins that exhibited H3K36 demethylase activity.^14^ Our previous study demonstrated that KDM2A was frequently overexpressed in breast tumor tissues and this demethylase upregulated Jagged1 to activate the Notch signaling pathway to promote cancer stemness and angiogenesis.^15^ Interestingly, we found that knockdown of KDM2A induced a significant increase of 5′-hmc level in genomic DNA suggesting a potential role of KDM2A in the regulation of DNA methylation. In this study, we tried to elucidate the underlying mechanism by which KDM2A regulates DNA methylation.

## Results and discussion

### KDM2A inhibits the expression of TET2 to increase DNA methylation

**Figure 1:**
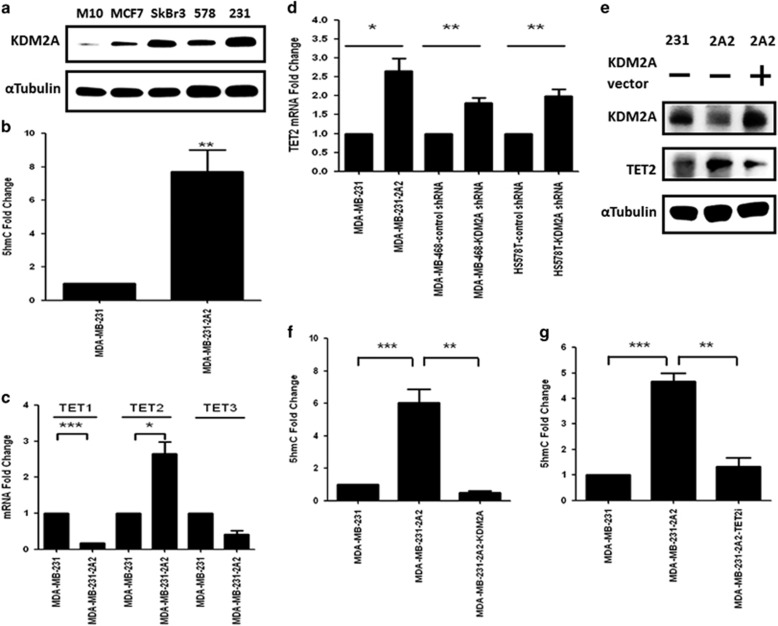
KDM2A inhibits the expression of TET2 to reduce the 5′-hmc level in breast cancer cells. (**a**) Cellular proteins were extracted from various breast cancer cell lines with a lysis buffer (50 mM Tris-HCl, pH 7.4, 150 mM NaCl, 1% NP-40, 0.1% sodium dodecyl sulfate (SDS), 0.5% sodium deoxycholate, 2 mM ethylenediaminetetraacetic acid (EDTA) and 50 mM sodium fluoride (NaF)) and the proteins were separated by SDS-polyacrylamide gel electrophoresis. Proteins were transferred to polyvinylidene difluoride (PVDF) membranes, probed with KDM2A antibody (Abcam, Cambridge, MA, USA) and the signal was developed by enhanced chemiluminescence reagent. α-Tubulin was used as an internal control. (**b**) Genomic DNA of MDA-MB-231 and KDM2A-depleted MDA-MB-231-2A2 cells was extracted by the Tissue & Cell Genomic DNA purification kit (GMbiolab Co. Ltd, Taiwan). The 5′-hydroxymethylcytosine (5′-hmc) level of genomic DNA was detected by using Quest 5-hmC TM DNA ELISA Kit (ZYMO Research Corp. Irvine, USA). Results from three independent assays were collected and the 5’-hmc level of MD-MB-231 cells was defined as 1. (**c**) Total RNA was isolated from cells, and 1 μg of RNA was reverse-transcripted to cDNA. Target mRNAs were quantified using real-time PCR reactions with SYBR green fluorescein and actin was served as an internal control. Primer sequences used for real-time PCR was showed in Supplementary Table 1. Data were shown as Mean±s.e.m. (**d**) MDA-MB-468 and Hs758T breast cancer cells were transfected with KDM2A shRNA and the mRNA level of TET2 was determined at 48 h after transfection. (**e**) MDA-MB-231 (231) or MDA-MB-231-2A2 (2A2) cells were transfected with control (—) or KDM2A expression vector. After 48 h, protein level of KDM2A and TET2 was studied by western blotting. (**f**) MDA-MB-231-2A2 cells were transfected with KDM2A expression vector and the 5′-hmc level of genomic DNA was determined by using Quest 5-hmC TM DNA ELISA Kit. (**g**) MDA-MB-231 or MDA-MB-231-2A2 cells were transfected without or with TET2 siRNA (Santa Cruz Biotechnology, Inc., USA), and the 5′-hmc level of genomic DNA was determined by using Quest 5-hmC TM DNA ELISA Kit. Statistical analysis was performed by using paired *t*-test and two-tailed *P*-values ⩽0.05 were considered statistically significant. ***P<0.001, **P<0.01, *P<0.05.

We screened the expression of KDM2A in a panel of breast cancer cell lines and found the upregulation of this demethylase in breast cancer cells when compared to that of M10 normal mammary epithelial cells (Figure 1a). Interestingly, three triple-negative breast cancer cell lines including MDA-MB-231, Hs-578T and MDA-MB-468 exhibited the highest expression of KDM2A (Figure 1a and data not shown). Therefore, we specifically focused on the study of KDM2A in triple-negative cells. Compared to the parental MDA-MB-231 cells, the KDM2A-depleted stable cell line (MDA-MB-231-2A2) exhibited an eightfold increase at the 5′-hmc level in the genomic DNA (Figure 1b). Because the expression of DNMTs was not significantly changed in MDA-MB-231-2A2 cells (data not shown), we investigated the expression of TETs and found that TET2 was significantly upregulated in the KDM2A-depleted cells (Figure 1c). This is not a cell line-specific effect because transient knockdown of KDM2A by shRNA also increased TET2 expression in the Hs-578T and MDA-MB-468 cells (Figure 1d). Ectopic expression of KDM2A reversed the upregulation of TET2 in the MDA-MB-231-2A2 cells suggesting TET2 is a direct repression target of KDM2A (Figure 1e). In addition, the 5′-hmc level was also reduced (Figure 1f). To verify the increase of 5′-hmc in the MDA-MB-231-2A2 cells was mediated by TET2, we inhibited the expression of TET2 by siRNA and confirmed the reduction of 5′-hmc in genomic DNA (Figure 1g). These results suggested KDM2A inhibits TET2 to increase DNA methylation.

### Repression of TET2 by KDM2A is RelA-dependent

**Figure 2:**
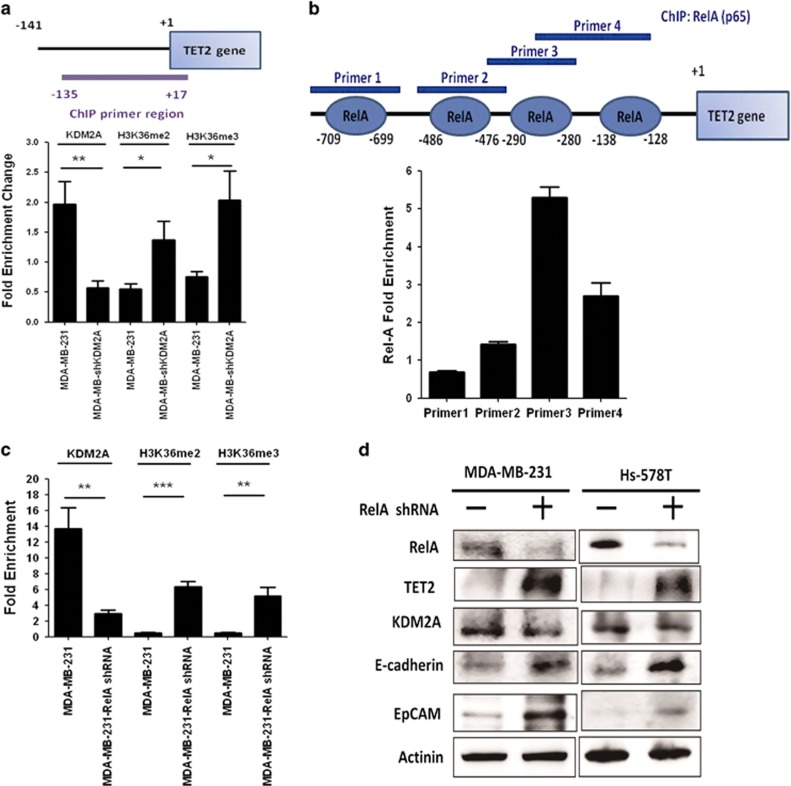
RelA is involved in the inhibition of TET2 by KDM2A. (**a**) The diagram in the upper panel showed the genomic region of human TET2 gene amplified in our ChIP assay. MDA-MB-231 cells were transfected with control (—) or KDM2A shRNA. After 48 h, cells were fixed with 1% formaldehyde at 37 °C for 10 min and washed twice with ice-cold PBS containing protease inhibitors. Cells were incubated in a lysis buffer (1% SDS, 10 mM EDTA, 50 mM Tris-HCl, pH 8.1) for 10 min on ice and sonicated to shear genomic DNA. The lysate was centrifuged for 10 min at 13 000 r.p.m. at 4 °C. The supernatant was diluted in a ChIP dilution buffer (0.01% SDS, 1% Triton X-100, 2 mM EDTA, 16.7 mM Tris-HCl, pH 8.1, 167 mM NaCl, and protease inhibitors). Anti-KDM2A, anti-dimethyl H3K36, anti-trimethyl H3K36 and non-immune (negative control) antibodies were added to the supernatant and incubated overnight at 4 °C with rotation. DNA fragments were recovered and subjected to PCR amplification. List of primer sequences used for ChIP assay was showed in Supplementary Table 1. (**b**) Transfection factor binding sites in the human TET2 gene promoter region were predicted by PROMO software (http://alggen.lsi.upc.es/) and the four potential RelA binding sites were shown in the upper panel. ChIP assay was carried out as described in (**a**) by using anti-RelA antibody (Thermo Fisher Scientific Inc., Waltham, MA, USA). The relative enrichment of RelA binding to the four potential sites was shown. (**c**) MDA-MB-231 cells were transfected with control or RelA shRNA. After 48 h, ChIP assay was conducted to investigate the binding of KDM2A to proximal TET2 gene promoter shown in (**a**). The methylation status (demethylation and trimethylation) of H3K36 in this region was also studied by ChIP assay. (**d**) MDA-MB-231 and Hs-578T cells were transfected with control (—) or RelA shRNA. The protein level of RelA, TET2 and KDM2A was studied by western blotting at 48 h after transfection.

KDM2A has been shown to be a H3K36 demethylase in cells.^16, 17^ Mechanistic study suggested that KDM2A utilized the zinc finger CxxC domain to recognize nonmethylated CpG dinucleotides in genomic DNA and catalyzed the demethylation of H3K36 proximal to the binding region via its enzymatic domain.^17^ Because the methylation of H3K36 in the promoters implied gene activation, the demethylation of this histone marker generally caused the downregulation of gene expression.^18, 19^ To confirm TET2 is a direct repression target of KDM2A, we performed chromatin immunoprecipitation (ChIP) assay to study the binding of KDM2A to the TET2 gene promoter. Our data showed that KDM2A constitutively bound to the TET2 promoter, and the depletion of KDM2A dramatically attenuated its promoter binding that was associated with the increase of di- and tri-methylation of H3K36 supporting KDM2A is an *in vivo* H3K36 demethylase as reported previously (Figure 2a).^16, 17^ Bioinformatics prediction suggested four RelA binding sites in the TET2 promoter region (Figure 2b). Two previous evidences promoted us to study the potential role of RelA in the regulation of TET2 expression by KDM2A. Firstly, RelA is constitutively activated in estrogen receptor-negative and triple-negative breast cancer cells.^20^ Secondly, a functional interaction between RelA and KDM2A has been reported recently.^21, 22^ We found that RelA constitutively bound to the human TET2 gene promoter and the two proximal sites upstream of the transcription start site showed the strongest binding (Figure 2b). This is consistent with the previous findings that RelA is constitutively activated, and binds to various gene promoters to stimulate or inhibit gene transcription in estrogen receptor-negative breast cancer cells.^20^ Because the most proximal RelA binding site located at the -138/-128 region overlapped with the KDM2A binding region detected in our ChIP study (Figure 2a), we hypothesized that RelA interacted with KDM2A and co-occupied at the proximal promoter region to repress gene transcription. Indeed, knockdown of RelA in the MDA-MB-231 cells significantly reduced the binding of KDM2A to the proximal promoter region of the TET2 gene (Figure 2c). In addition, the di- and tri-methylation of H3K36 at this region was increased. We also confirmed the depletion of RelA in the MDA-MB-231 and Hs-578T cells restored the expression of TET2 as found in KDM2A-depleted MDA-MB-2A2 cells (Figure 2d). These data suggested that RelA and KDM2A form a repression complex to demethylate H3K36 in the promoter region to attenuate TET2 transcription. Previous studies demonstrated that KDM2A acts as a negative regulator of RelA by demethylating the K218 and K221 residues.^21, 22^ However, it should be noted that six lysine residues and one arginine residue of RelA have been shown to be methylated *in vitro* and *in vivo*.^23^ The biological outcome elicited by different combinations of these methylations is more complex than originally proposed and needs further characterization. Here, we provide another model that RelA acts as an anchor protein that constitutively binds to the TET2 promoter and may recruit KDM2A to demethylate H3K36 to attenuate TET2 expression when KDM2A is overexpressed.

### Increase of TET2 expression induced by KDM2A depletion promotes the re-activation of the downstream target genes to suppress cell invasiveness

**Figure 3:**
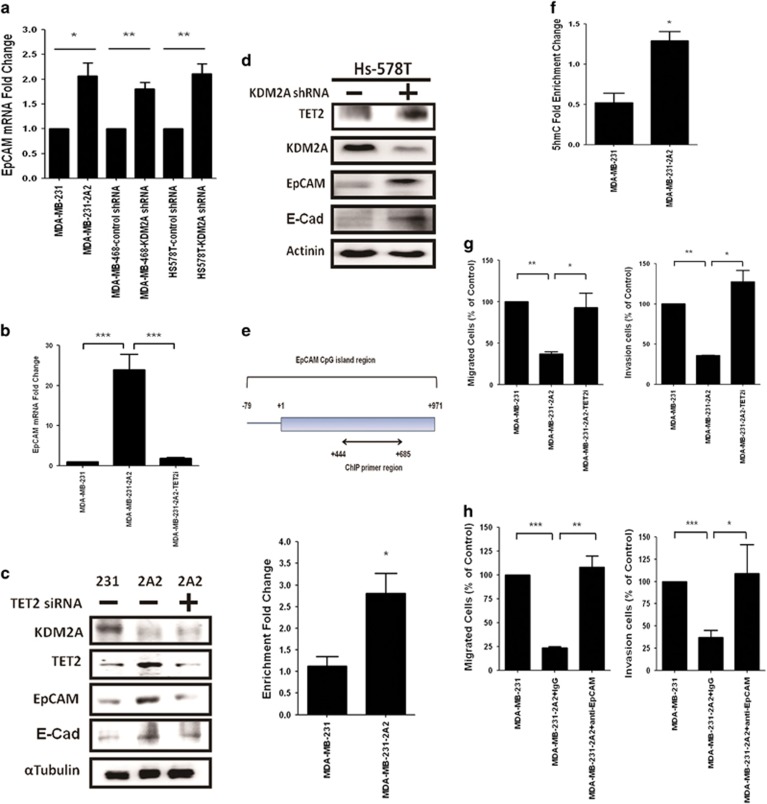
Increase of TET2 induced by KDM2A depletion re-activates the downstream target genes and attenuates cell invasiveness. (**a**) MDA-MB-231, MDA-MB-468 and Hs-578T cells were transfected with control (—) or KDM2A shRNA. After 48 h, the expression of EpCAM was studied by real-time RT–PCR and was compared between control and KDM2A-depleted cells. (**b**) MDA-MB-231-2A2 cells were transfected without or with TET2 siRNA and the expression of EpCAM was investigated by real-time RT–PCR at 48 h after transfection. (**c**) Protein level of two reported TET target genes EpCAM and E-cadherin in MDA-MB-231 cells and MDA-MB-231-2A2 cells transfected without or with TET2 siRNA was also studied by western blotting. (**d**) Hs-578T cells were transfected with control (—) or KDM2A shRNA. After 48 h, protein level of EpCAM and E-cadherin was investigated. (**e**) The upper diagram showed the prediction of a CpG island (−79 to +971) in human EpCAM promoter by the University of California Santa Cruz genome browser (https://genome.ucsc.edu) and the region (+444 to +685) amplified by our PCR primer was shown. The binding of TET2 to this CpG region in MDA-MB-231 and MDA-MB-231-2A2 cells was studied by ChIP assay. (**f**) The 5′-hmc level in the amplified region was also investigated by using anti-5′-hmc antibody for ChIP assay. (**g**) MDA-MB-231 or MDA-MB-231-2A2 cells were transfected without or with TET2 siRNA. Migration assays were carried out in transwells with 5-μm pore filter inserts on 24-well plates. For invasion assays, the transwell inserts were coated with gelatin A/B solution before the cells were seeded. The lower chamber was filled with medium containing 1% serum. After 12 h, the filter was gently removed from the chamber, the cells on the upper surface were removed by wiping with a cotton swab, and cells that migrated to the lower surface areas were fixed, stained with DAPI and counted in 15 randomly selected fields in a microscope. Experiments were repeated three times. (**h**) MDA-MB-231 and MDA-MB-231-2A2 cells were incubated with non-immune IgG or anti-EpCAM antibody and subjected to migration and invasion assays as described in (**f**). **P<0.01, *P<0.05.

To characterize the biological consequences of the upregulation of TET2 induced by KDM2A depletion, we investigated the expression of two reported TET target genes EpCAM and E-cadherin. In the MDA-MB-231-2A2 cells, the expression of EpCAM was significantly increased (Figure 3a). Upregulation of EpCAM was also found in the Hs-578T and MDA-MB-468 cells transfected with the KDM2A shRNA. The enhancement of EpCAM expression was dependent on TET2 because knockdown of TET2 abolished the induction (Figure 3b). The protein level of EpCAM and E-cadherin in the MDA-MB-231-2A2 cells and the KDM2A-depleted Hs-578T cells was also increased (Figures 3c and d). Similarly, knockdown of RelA which induced the upregulation of TET2 also increased EpCAM and E-cadherin proteins (Figure 2d). Bioinformatics prediction suggested a CpG island located within the −79/+971 region of the human EpCAM gene (Figure 3e). Our ChIP assay demonstrated that depletion of KDM2A enhanced the binding of TET2 to this CpG island (Figure 3e), and the 5′-hmc level in this region was also increased (Figure 3f) supporting our hypothesis that KDM2A depletion upregulated TET2 expression to induce the demethylation of the EpCAM promoter to activate its gene transcription. EpCAM and E-cadherin are involved in the control of cell–cell contact and invasiveness. We therefore studied the alteration in migration and invasion and found that the depletion of KDM2A reduced cell migration and invasion which could be reversed by knockdown of TET2 (Figure 3g). Inhibition of EpCAM by blocking antibody also attenuated cell invasiveness (Figure 3h). These data suggested that KDM2A depletion increases TET2 and re-activates TET2 downstream target genes to suppress cell invasiveness.

### TET2 expression is inversely correlated with KDM2A and predicts a better survival in the triple-negative breast cancer patients

**Figure 4:**
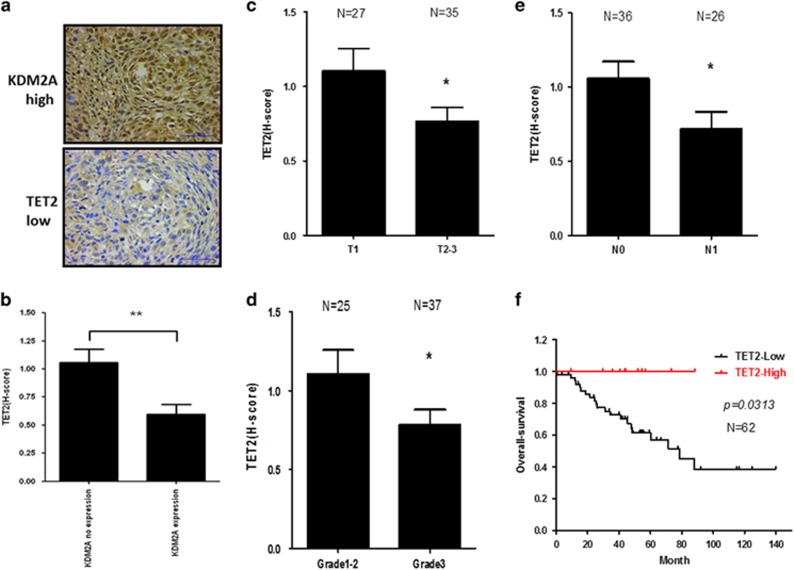
TET2 expression is negatively associated with KDM2A and predicts a better survival in triple-negative breast cancer patients. (**a**) Typical immunohistochemical staining showed strong KDM2A and low TET2 expression in a tumor tissue. Paraffin-embedded tissue sections of 62 human triple-negative breast cancer specimens were obtained from Department of Pathology, Kaohsiung Medical University Hospital (Kaohsiung, Taiwan). The slides were stained with anti-KDM2A and anti-TET2 antibody and the staining was interpreted using the H-score, defined by the following equation: H-score=ΣPi (i + 1) as previously described.^15^ Institutional review board approval for using these human tissues in this study was given by the Research Ethics Committee of the Kaohsiung Medical Hospital (IRB: KMUHIRB-E(II)-20150086). (**b**) The association between the expression of KDM2A and TET2 was compared. In addition, the expression of TET2 in patients with different tumor sizes (**c**), grade (**d**) and lymph node metastasis (**e**) was compared. (**f**) Patient’s overall survival was compared by the Kaplan–Meier plots and compared using the log-rank test. **P<0.01, *P<0.05.

Because KDM2A was expressed at the highest level in triple-negative breast cancer cell lines, we investigated the association of KDM2A and TET2 in 62 triple-negative breast tumor tissues. Figure 4a demonstrated the tumor tissue of a patient with strong KDM2A staining showed very low expression of TET2. Statistical analysis showed an inverse correlation between TET2 and KDM2A in the tumor tissues (Figure 4b). In addition, downregulation of TET2 was associated with advanced stage (Figure 4c), lymph node metastasis (Figure 4d) and high tumor grade (Figure 4e). More importantly, TET2 expression predicted a better survival in the patients (Figure 4f). A recent study demonstrated that the decrease of global 5′-hmc was associated with poor disease-specific and disease-free survival in breast cancer patients.^24^ However, the mechanism of the reduction of 5′-hmc level and TET expression was not addressed. Here, we provide the first evidence that KDM2A is an upstream inhibitor of TET2, and may modulate the global 5′-hmc by suppressing TET2. When our study was undergoing, Borgel *et al.*^25^ reported the potential role of KDM2A in the mediation of DNA methylation and gene silencing. The working hypothesis proposed in the study is that KDM2A directly interacts with heterochromatin protein 1 (HP1) via its zinc finger CxxC and PHD domains to form a nucleosome binding circuit to recruit the H3K9 lysine methyltransferases to introduce H3K9 methylation. In addition, HP1 also recruited DNMTs to deposit CpG methylation. The overall result is the simultaneous increase of H3K9 and CpG methylation which leads to the establishment of heterochromatin. Our results establish a new model that differs from the previous model in two ways. Firstly, the major effect of KDM2A on DNA methylation is mediated by the recruitment of DNMTs in the Borgel’s study. However, alteration of the expression of DNA demethylases and methyltransferase was not studied. We identified TET2 is a repression target of KDM2A and is important for the modulation of DNA methylation by KDM2A. Secondly, the main histone marker investigated in the Borgel’s study is H3K9. On the contrary, we found a global change of the 5′-hmc level and H3K36 methylation in different gene promoters after KDM2A depletion indicating KDM2A has a board impact on the coupling of DNA and histone methylation to regulate gene expression. Collectively, this study reveals a novel mechanism by which the methylation of DNA and histone is co-regulated via modulating the expression of TET2 by KDM2A.

## Publisher’s note

Springer Nature remains neutral with regard to jurisdictional claims in published maps and institutional affiliations.
