# Supplementary material for: Lysine demethylase KDM2A inhibits TET2 to promote DNA methylation and silencing of tumor suppressor genes in breast cancer
Source: Oncogenesis. 2017 Aug 7;6(8):e369–. doi: 10.1038/oncsis.2017.71 (PMC5608919; doi:10.1038/oncsis.2017.71)
Supplement: Supplementary Table 1 [file oncsis201771x1.pdf]

Supplementary Table 1. The sequence of the primers used in this study

**RT-PCR**	
TET1-Forward	5’-CCTTTGGGAACCGACTCCTC-3’
TET1-Reverse	5’-CACAAGGTTTTGGTCGCTGG-3’
TET2-Forward	5’-CTCGCATGCAAGTCACGTCC-3’
TET2-Reverse	5’-ATGTTTGCCAGCCTCGTTCT-3’
TET3-Forward	5’-ACCTGCCAGGCCTTTATGAC-3’
TET3-Reverse	5’-CCAGCCTTTATTTCCACCTCCT-3’
EpCAM-Forward	5’-TTGCTCAAAGCTGGCTGCCA-3’
EpCAM-Reverse	5’-GGATCCAGTTGATAACGCGT-3’
ChIP primer	
TET2-ChIP-Fowrward	5’-GGGCTCTTACGAGAGGCAAC-3’
TET2-ChIP-Reverse	5’-CGGCCCACCTTCTGTTTACT-3’
EpCAM-ChIP-Forward	5’- GGATTGGAGCAGAGTTGTG-3’
EpCAM-ChIP-Reverse	5’- AAGGCCATTTCCCTACCAAG-3’
TET2-ChIP p65-1- Forward	5’- CTTCTTAGCAACAATGCGACA-3’
TET2-ChIP p65-1- Reverse	5’- TATGGGATCACTGCATGGTG-3’
TET2-ChIP p65-2- Forward	5’- CACCATGCAGTGATCCCATA-3’
TET2-ChIP p65-2- Reverse	5’- TTCCCCCTTCTAATGCTCCT-3’
TET2-ChIP p65-3- Forward	5’- TGGGCTTTGTTCTTCATCTCA-3’
TET2-ChIP p65-3- Reverse	5’- GAAACCGACCGATACAGAGC-3’
TET2-ChIP p65-4- Forward	5’- TCAGGGAAAATCAAGCATCC-3’
TET2-ChIP p65-4- Reverse	5’- ACCCCCTACTTCTCGCTAGG-3’
